# The Endometrial Microbiota—16S rRNA Gene Sequence Signatures in Healthy, Pregnant and Endometritis Dairy Cows

**DOI:** 10.3390/vetsci10030215

**Published:** 2023-03-10

**Authors:** Anne A. M. J. Becker, Stacie Munden, Evonne McCabe, Daniel Hurley, Séamus Fanning, Aspinas Chapwanya, Patrick Butaye

**Affiliations:** 1 Department of Biomedical Sciences, Ross University School of Veterinary Medicine, Basseterre, Saint Kitts and Nevis; 2 Science Center South, School of Public Health, Physiotherapy & Sports Science, University College Dublin Belfield, D04 N2E5 Dublin, Ireland; 3 Department of Clinical Sciences, Ross University School of Veterinary Medicine, Basseterre, Saint Kitts and Nevis; 4 Department of Pathobiology, Pharmacology and Zoological Medicine, Faculty of Veterinary Medicine, Ghent University, 9820 Merelbeke, Belgium

**Keywords:** uterine microbiome, bovine endometritis, post-partum uterine disease, 16S rRNA gene amplicon-based metagenomics

## Abstract

**Simple Summary:**

In pursuit of a consistent diagnosis, treatment, and prevention of uterine diseases in dairy cattle, researchers have focused their efforts on identifying bacteria present in the uterus, the so-called uterine microbiota. Bacteria frequently populate the uterus but an optimal balance in type and number of bacteria is important for reproductive health. In this study, the uterine microbiota from healthy, pregnant, and diseased cattle have been analyzed from samples taken in the slaughterhouse. Interestingly, the bacterial composition in the uterus of healthy and pregnant cattle was more similar, compared to diseased animals who had a different bacterial composition in their uterus.

**Abstract:**

Endometritis is one of the most important causes of infertility in dairy cows, resulting in high economic losses in the dairy industry. Though the presence of a commensal uterine microbiota is now well established, the complex role of these bacteria in genital health, fertility, and susceptibility to uterine diseases remains unclear. In this study, we explore the endometrial microbiota through 16S rRNA gene profiling from cytobrush samples taken ex vivo from healthy, pregnant, and endometritis cows. There were no significant differences between healthy and pregnant cows, whose uterine microbiota were dominated by *Streptococcus*, *Pseudomonas*, *Fusobacterium*, *Lactococcus* and *Bacteroides*. Compared to pregnant and clinically healthy cows, the uterine bacterial community of endometritis cows was significantly decreased in species diversity (*p* < 0.05), reflecting uneven community composition in different patterns with either dominance of *Escherichia-Shigella*, *Histophilus*, *Bacteroides* and *Porphyromonas* or Actinobacteria.

## 1. Introduction

Bovine postpartum uterine diseases (PPUDs) are a common problem in dairy herds and a major concern for dairy farmers [1]. They are complex entities that comprise several (sub)clinical appearances, each one with a different degree of severity, which affect the reproductivity and consequentially decrease the profitability. Moreover, the treatment of PPUDs with antimicrobials is very costly, estimated between $344 and $410 per animal when compared to non-affected cows, including amongst others also losses of milk and feed cost [2]. Antimicrobials also select for antimicrobial resistant bacteria. The causes of uterine disease are multifactorial and dependent on the intricate balance between host immunity and the pathogenicity of the bacteria contaminating the uterus after parturition [3].

Over the last decade, studies have reported on the potential association between microbial infections, poor reproductive outcomes, and endometritis, particularly chronic endometritis [4]. To date, the main pathogens involved in the clinical manifestation of uterine diseases (endometritis, metritis, and pyometra) have been characterized and include *Escherichia coli*, *Trueperella pyogenes*, *Fusobacterium necrophorum*, *Bacteroides* spp., and *Prevotella* spp. [5,6,7]. Additionally, numerous opportunistic pathogens such as *Staphylococcus* spp., *Enterococcus* spp., *Streptococcus* spp., and *Bacillus* spp. have been isolated [7,8,9]. The findings of these culture-dependent studies have recently been corroborated and expanded by 16S rRNA amplicon-based sequencing. These 16S rRNA gene-based studies reveal a larger intrauterine bacterial population diversity and a more detailed and complex structure of the uterine microbiota in both metritis and healthy cows [6,10,11,12,13]. Moreover, the finding of bacteria in the uterus of virgin heifers and pregnant cows challenges the hypothesis that a sterile uterus is required for the establishment and maintenance of pregnancy [7,14,15]. In humans, the importance of the uterine microbiota for endometrial physiology, reproductive health, and fertility has increasingly been recognized [16]. Commensal microbiota may convey protection towards pathogenic species, prime the uterine immune response, and influence endometrial receptivity before pregnancy [17]. It has been shown that dairy cows have an established uterine microbiome before calving and its structure remains identical between cows that develop metritis and healthy cows until 2 days postpartum. Thereafter, cows that develop metritis experience a shift in the uterine microbiome, characterized by a loss of heterogeneity and a reduced richness [18]. Evaluating the uterine microbial diversity at time of diagnosis is valuable. Further, it will also be informative to investigate the changes in the uterine microbiota before diagnosis of uterine disease, and to compare this with the uterine microbial composition in healthy and pregnant cows. The latter hold in the uterus throughout gestation, with bacteria belonging to the families Porphyromonadaceae, Ruminococcaceae and Lachnospiraceae with some potentially pathogenic species, though their presence was not associated with inflammation [19]. Characterization of endometrial microorganisms is essential for understanding how a host evolves in association with its microbial symbionts, diagnosing disease, and exploring the origins of perturbed fertility in cattle. To this end, this study aims to specifically characterize the microbiota present in the endometrium of healthy, pregnant, and endometritis dairy cows.

## 2. Materials and Methods

### 2.1. Sample Collection

Endometrial swabs were collected from Holstein Friesian dairy cows, slaughtered for commercial reasons, ex vivo (*n* = 26) at a slaughter facility in Co. Kildare, Ireland. The animals were brought to the slaughterhouse by different farmers. No further background information and metadata on the cows is available. Animals were selected based on the clinical appearance of the ovaries and uterine contents after evisceration. The phase of the cycle was assessed in all animals. Each animal had a CL on the ovary (pregnant or in luteal phase for the nonpregnant). Therefore, all the animals were under the influence of progesterone. None of the animals had developed placentomes [20], indicating that they were in the early stage of gestation.

Sample size was calculated using a one-sample *t* test power calculation with *n* = 5, = 0.5, significance level = 0.05, power = 0.2389952, alternative = greater. Six to ten animals were planned to be selected per group. The animals were divided into three groups: healthy (*n* = 10) if no signs of endometritis were present; pregnant (*n* = 10) if an embryo was present and no placentomes; diseased (*n* = 6) if the uterus contained purulent exudate. To obtain endometrial samples, each genital tract was placed on a disinfected aluminum foil before sampling. Individually wrapped and sterilized surgical instruments were used for each tract. The uterus was opened aseptically, and endometrial contents sampled using an Orcellex cytobrush by rotating and rolling the brush on the mucosa surface. Two samples were taken and each brush was then placed into 1 mL sterile tubes, transported on dry ice, and stored at −80 °C. The first cytobrush was kept until DNA extraction. The second cytobrush was used to prepare endometrial smears for cytological assessment and classification of the animals [21,22,23,24]. Endometritis animals had 18% or greater neutrophils on a cytological assessment.

### 2.2. DNA Extraction

The brush tips were removed and placed into a filtered homogenizer bag. Ten milliliters of phosphate buffered saline (PBS, Sigma-Aldrich, St Louis, MO, USA) was added to each bag containing the brush tips and homogenized for 3 min. After homogenization, each sample was transferred to a 50 mL Falcon tube and centrifuged at 10,000× *g* for 10 min. The supernatant was discarded, and the pellet was re-suspended in 550 µL of 1X TE (Tris-EDTA pH8.0) buffer and transferred to a 2 mL Eppendorf tube, then 30 µL of 10% [*w*/*v*] SDS (sodium dodecyl sulphate) and 20 µL of 20 mg/mL proteinase K were added to each sample tube and vortexed for 5 s and incubated at 45 °C for 1 h in a water bath. After 1 h, 100 µL of 5 M NaCl, 80 µL of CTAB/NaCl (10% [*w*/*v*] Cetyl trimethylammonium bromide/0.7 M NaCl) solution was added to each sample and vortexed and incubated for 10 min at 65 °C. After 10 min, an equal volume of phenol chloroform (Sigma–Aldrich) was added to each sample, vortexed, and centrifuged at 10,000 rpm for 5 min. After centrifugation, the aqueous phase was transferred to a fresh 2 mL Lo-bind Eppendorf tube and an equal volume of phenol chloroform isoamyl alcohol (P:C:IA in 25:24:1, Sigma–Aldrich) was added, vortexed, and centrifuged at 10,000× *g* for 10 min and then the aqueous phase was once again transferred to a fresh 2 mL Lo-bind Eppendorf tube.

DNA was precipitated by adding 0.6× volume of isopropanol and stored at −20 °C overnight, which was followed by centrifugation at 10,000 rpm for 5 min. The DNA pellet was washed finally with 1 mL of 70% [*v*/*v*] alcohol and centrifuged at 10,000 rpm for 5 min and dried in a vacuum dryer. The dried DNA pellet was dissolved in 100 µL of 1X TE buffer. The concentration and purity of the DNA was determined on a Nano Drop™ spectrophotometer measuring absorbance at 260 nm and the ratio of absorbance at A260/A280.

### 2.3. 16S rRNA Gene Amplification and Sequencing

Libraries were prepared for all 26 samples as outlined in the 16S Metagenomic Sequencing Library preparation for the Illumina MiSeq system (Part # 15044223 Rev. B). The 16S rRNA V3–V4 hypervariable regions were amplified using the S-D-Bact-0341-b-S-17 (5′-CCTACGGGNGGCWGCAG-3′) and S-D-Bact-0785-a-A-21 (5′-GACTACHVGGGTATCTAATCC-3′) primers [25]. Amplicons for each sample template DNA were generated by setting up the following reaction in lo-bind (Eppendorf) 96 well plate with 2.5 µL genomic DNA (5 ng/µL), 5 µL amplicon PCR forward primer, 5 µL amplicon PCR reverse primer and added to 12.5 KAPA HiFi HotStart ReadyMix, gently mixed, and centrifuged for 1000× *g* for 1 min. Amplification was performed in a thermal cycler using the following conditions: 95 °C for 3 min and 25 cycles of 95 °C for 30 s, 55 °C for 30 s, 72 °C for 30 s, followed by annealing at 72 °C for 5 min. Each amplicon was assessed on the Agilent 2200 TapeStation using Agilent D1000 reagents and ScreenTape (Agilent Technologies, Santa Clara, CA, USA) to verify size, the expected size being ~550 bp. After the size had been verified, each amplicon underwent purification using AMPure XP beads to purify the V3 and V4 amplicon from primers and primer dimers. Once the amplicons were purified, dual indices and Illumina sequencing adaptors were added using the Nextera XT index kit. In a lo-bind 96 well plate, 5 µL of amplicon DNA, 5 µL Nextera XT index primer 1 (N7), 5 µL Nextera XT index primer 2 (S5), 25 µL 2 × 5 KAPA HiFi HotStart ReadyMix, and 10 µL of PCR grade water were added, gently mixed, and centrifuged for 1000× *g* for 1 min. PCR was performed in a thermal cycler using the following conditions: 95 °C for 3 min and 8 cycles of 95 °C for 30 s, 55 °C for 30 s, 72 °C for 30 s, followed by annealing at 72 °C for 5 min. The Indexed PCR samples were then purified as before and 1:10 dilution of each library preparation was assessed once again on the Agilent 2200 TapeStation using Agilent D1000 reagents and ScreenTape (Agilent Technologies, Santa Clara, CA, USA) to verify the fragment size (expected fragment size ~630 bp). DNA concentration of each sample library preparation was determined using the Qubit dsDNA Assay Kit (Invitrogen, Waltham, MA, USA).

The average fragment lengths and Qubit DNA concentration were used to normalize the DNA to a final concentration of 4 nM. All 20 of the 4 nM normalized samples were then pooled by combining 5 µL of each. From this normalized pooled, a volume of 5 µL was denatured by adding it to 5 µL 0.2 M NaOH and incubating for five minutes. After denaturation, 0.2 M Tris-HCl (pH 8.0) was added to neutralize the NaOH and subsequently diluted using 985 µL HT1 buffer from the MiSeq^®^ Reagent Kit v3 to a final concentration of 20 pM. The 20 pM library was further diluted by adding 300 µL of this library to 300 µL of HT1 buffer and mixed to give a library with a final concentration of 10 pM. Denatured PhiX with a concentration of 20 pM (30 µL) was added to 570 µL of the denatured 10 pM library to give a final spike of 5% of the library. The library was subsequently incubated at 95 °C for two min and placed on ice. After five min, 600 μL of the 20 pM library was loaded onto a reagent cartridge from the MiSeq^®^ Reagent Kit v3, which was loaded onto the Illumina^®^ MiSeq Instrument for 600 cycles of paired-end sequencing.

The quality of the generated reads was assessed using FastQC (version 0.11.8). Raw sequencing reads were then demultiplexed, quality-filtered, and analyzed using Mothur (version v.1.43.0) [26]. Sequences were aligned against SILVA (release 132) reference database [27]. Chimeras were checked and removed using the VSEARCH algorithm (version v2.13.3) [28]. The resulting high-quality amplicons were clustered into operational taxonomic units (OTUs) with a 97% similarity threshold in Mothur.

### 2.4. Data Analysis

Downstream analyses were carried out in RStudio (v1.3.1093, [29]) using the packages vegan (v.2.5-7), WGCNA, ggplot2 (v.3.3.3), phyloseq (v.1.34.0), pairwiseAdonis (v.0.0.1), and microbiome (v.1.12.0). The bacterial phylogenetic core of the uterine samples from healthy cows was analyzed using the microbiome package (v1.12.0) and to identify core bacterial genera, we set the prevalence threshold in our dataset to 50% with a detection threshold of 0.1%. Alpha diversity measures (Observed Species and Shannon Diversity Index) were analyzed between groups using Kruskal–Wallis tests and pairwise Wilcoxon tests (alpha = 0.05). For beta diversity, principal coordinate analysis based on Bray Curtis dissimilarity metrics was used to assess differences in uterine bacteria composition by condition (healthy, pregnant, endometritis) and the outcome evaluated using non-parametric multivariate analysis of variance (PERMANOVA). Venn diagrams were generated to show the number of shared OTUs among healthy, pregnant and endometritic cows. Significant differences in the abundance of OTUs were identified using a multifactorial negative binomial GLM, implemented in the R package DESeq2 (v.1.13.1). Significantly different OTUs (*p*-value adjusted by FDR < 0.1) between healthy cows, endometritic, and pregnant cows were determined using the Wald test for significance of GLM terms.

## 3. Results

Of the 26 animals sampled, two were excluded from further analysis based on poor-quality sequences, leaving ten healthy, ten pregnant, and four endometritis based on clinical evaluation. Pregnancy of the animals was early gestation (less than 42–56 days) since the placenta had not yet developed [30]. After cytological examination, one clinically healthy animal was reclassified as diseased. Thus, cytological assessment grouped the animals into healthy (*n* = 9), pregnant (*n* = 10), and endometritis (*n* = 5) animals (18% or greater neutrophil count on cytological assessment).

After filtering out low quality reads, a total of 2,275,115 reads were obtained with 94,796 ± 86,440 reads per sample on average. The average read length was 288 bp (min = 267 bp, max = 300 bp). High-quality reads were sorted into 7740 operational taxonomic units (OTUs) using a 97% sequence identity cut off. Singletons were removed for analyses, resulting in a dataset totalling 3188 OTUs. On average, we detected 355 ± 252 OTUs per sample.

### 3.1. Taxonomic Composition of the Uterine Bacterial Community in Clinically Healthy Dairy Cows (n = 9)

**Figure 1:**
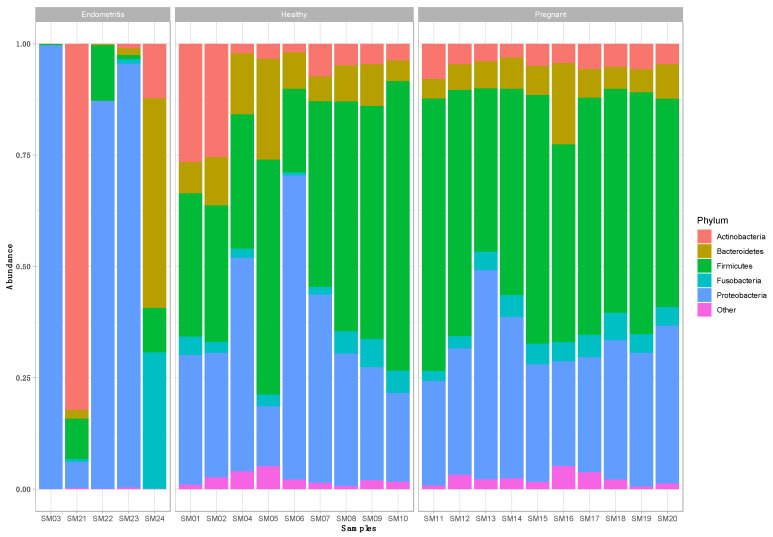
Relative abundance of phylotypes at the phylum level in endometrial cytobrush samples (SMx) taken ex vivo from healthy (“healthy”; *n* = 9) and pregnant cows (“pregnant”; *n* = 10), and cows diagnosed with endometritis (“diseased”; *n* = 5).

From the nine healthy animals, a total of 2157 OTUs were retrieved, with an average Shannon index of 4.41 ± 0.40 and 290 ± 122 OTUs per animal. Phyla, whose relative abundance was less than 1% of total reads, were grouped as “Other”, resulting in five phyla that comprised 97.09% of the total dataset. Proteobacteria (45.91%), Firmicutes (33.02%), and Bacteroidetes (12.11%) were predominant, followed by a minority of Actinobacteria (4.23%) and Fusobacteria (1.82%) (Figure 1).

Within the predominant phylum Proteobacteria, a little more than half of the reads were assigned to the Enterobacteriaceae (51.32%), largely represented by the genera *Escherichia*-*Shigella* (12.21%) and a group of unclassified Enterobacteriaceae (83.77%), followed by the Pseudomonadaeae (13.74%) with *Pseudomonas* as the main genus. Other families included Neisseriaceae (9.29%), Pasteurellaceae (8.31%), Burkholderiaceae (5.40%), and Moraxellaceae (3.95%).

Within the phylum Firmicutes, approximately a quarter (26.24%) of the reads were assigned to the genus *Streptococcus*, belonging to Streptococcaceae (Lactobacillales; Bacilli), which was the predominant family (30.48%). The Lachnospiraceae (Clostridiales; Clostridia) were the second most abundant family (22.60%) in this phylum, largely represented by the genus *Blautia* (8.42%) and *Acetitomaculum* (2.82%). Another fourth of the reads were assigned to Ruminococcaceae (7.93%), Bacillaceae (5.85%), Veillonellaceae (4.34%), Erysipelotrichaceae (3.84%), and Staphylococcaceae (3.30%).

Within the phylum Bacteroidetes, Prevotellaceae (30.37%) and Bacteroidaceae (30.24%) accounted for more than half of the reads, followed by Porphyromonadaceae (22.24%). The phylum Actinobacteria was represented by different families at comparable relative abundances, such as Microbacteriaceae (20.26%), Micrococcaceae (15.79%), Corynebacteriaceae (14.35%), Actinomycetaceae (13.88%), and Propionibacteriaceae (12.46%). Finally, the phylum Fusobacteria comprised two families, Fusobacteriaceae (74.54%), represented by the genus Fusobacterium and Leptotrichiaceae (25.46%).

**Figure 2:**
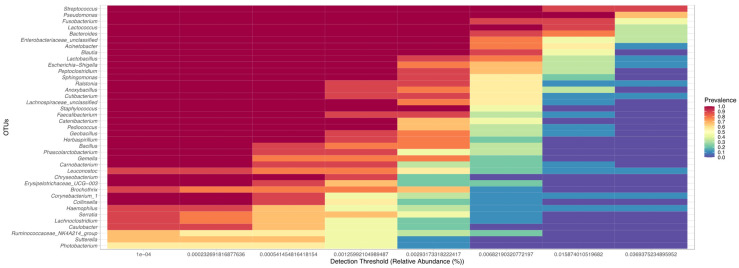
Heatmap of the core microbiome analysis to identify at genus level core taxa in the endometrium of clinically healthy dairy cattle (*n* = 9). The y-axis represents the prevalence level of core genera across the detection threshold (relative abundance) range on x-axis. The variation of prevalence of each genus is indicated by a gradient of color from blue (decreased) to red (increased). Only the genera with minimum prevalence of 0.5 (50%) at 0.001 (0.1%) abundance are plotted.

At a detection threshold set at 0.1%, 39 genera were detected to be present in >50% of the uterine brush samples from healthy cows. The top five taxa shared included *Streptococcus*, *Pseudomonas*, *Fusobacterium*, *Lactococcus*, and *Bacteroides* (Figure 2).

### 3.2. Taxonomic Composition of the Uterine Bacterial Community in Dairy Cows with Endometritis (n = 5)

From five endometritis animals, a total of 928 OTUs were retrieved, with an average Shannon index of 0.95 ± 0.69 and 285 ± 172 OTUs per animal. Phyla whose relative abundance was less than 1% of total reads were grouped as “Other”, resulting in four phyla that comprised 94.39% of the total dataset. The relative abundance of Proteobacteria was the highest at 67.25%, followed by Actinobacteria (11.49%), Bacteroidetes (8.65%), and Firmicutes (7.00%). However, taxonomic profiles of the samples were markedly different between the five diseased animals (Figure 1).

In three animals, Proteobacteria dominated with Escherichia-Shigella (Enterobacteriaceae; Enterobacteriales; Gammaproteobacteria) accounting for a relative abundance of 87.13% and 95.20%, respectively, in animals SM22 and SM23, both showing clinical signs. In contrast, the genus *Histophilus* (Pasteurellaceae; Pasteurellales; Gammaproteobacteria) prevailed in the subclinical diseased animal SM03, with a relative abundance of 99.66%. In contrast, Proteobacteria only contributed marginally to the taxonomic profile of the other two diseased animals SM21 and SM24, at a relative abundance of 5.83% and 0.09%, respectively. The taxonomic profile for animal SM21 was dominated by Actinobacteria (82.21%), with 90% of reads assigned to unclassified Microbacteriaceae and only a small contribution of Firmicutes (9.04%) and Bacteroidetes (1.99%). However, for animal SM24, Bacteroidetes dominated the taxonomic profile with a relative abundance of 47.08%, represented mainly by the genera *Porphyromonas* (Porphyromonadaceae; Bacteroidales; 51.06%) and *Bacteroides* (Bacteroidaceae; Bacteroidales; 48.91%). Actinobacteria contributed for 12.25% whereas 30.59% belonged to other phyla. Among those, the genus Fusobacterium belonging to the phylum Fusobacteria accounted for 89.67%.

### 3.3. Taxonomic Composition of the Uterine Bacterial Community in Pregnant Dairy Cows (n = 10)

From 10 endometrial cytobrush samples from pregnant cows, a total of 1274 OTUs were retrieved, with an average Shannon index of 4.39 ± 0.40 and 285 ± 118 OTUs per animal. Phyla whose relative abundance was less than 1% of total reads were grouped as “Other”, resulting in five phyla that comprised 97.85% of the total dataset. Firmicutes (52.50%) and Proteobacteria (29.63%) were predominant, followed by a minority of Bacteroidetes (6.36%), Actinobacteria (5.24%), and Fusobacteria (4.12%) (Figure 1).

Within the Firmicutes, Streptococcaceae (41.21%) were dominant and represented by the genera *Streptococcus* (71.09%) and *Lactococcus* (28.91%). Other families included Lachnospiraceae (12.45%), Bacillaceae (8.30%), Lactobacillaceae (7.95%), Erysipelotrichaceae (7.85%), and Ruminococcaceae (5.26%). The Proteobacteria were largely represented by Pseudomonadaceae (23.17%), Enterobacteriaceae (19.02%), Moraxellaceae (16.93%), Sphingomonadaceae (7.28%), Pasteurellaceae (7.21%), and Neisseriaceae (5.20%). Within the Bacteroidetes, the relative abundance of Prevotellaceae accounted for 22.13%, followed by Weeksellaceae (6.57%) and Bacteroidaceae (5.68%). The Actinobacteria were largely composed of Propionibacteriaceae (31.51%), Micrococcaceae (25.70%), and Corynebacteriaceae (18.96%).

### 3.4. Comparison of the Uterine Bacterial Composition between Pregnant, Clinically Healthy and Endometritic Cows

**Figure 3:**
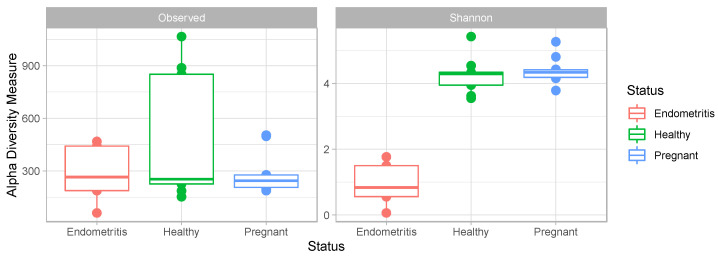
Boxplot of observed richness (number of OTUs) and Shannon Diversity Index in different groups. The boxes denote interquartile ranges (IQR) with the median as a line and whiskers extending up to the most extreme points within 1.5-fold IQR.

**Figure 4:**
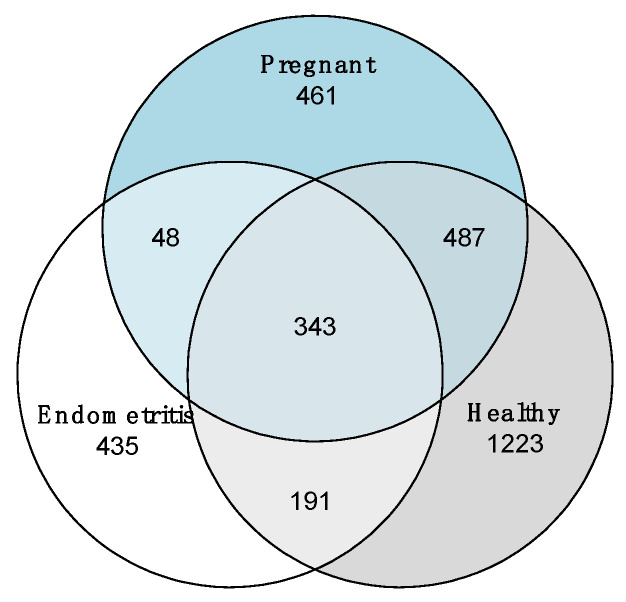
A Venn diagram showing the number of shared and unique OTUs at 97% identity among the three groups: healthy cows (*n* = 9), pregnant cows (*n* = 10), and cows diagnosed with endometritis (*n* = 5).

The uterine bacterial composition did not differ significantly between the pregnant and healthy animals. However, compared to pregnant and clinically healthy cows, the uterine bacterial community of endometritic cows was significantly decreased in species diversity (measured by Shannon index; X^2^ = 8.932, *p* < 0.05), though no significant differences were found among groups in species richness (measured by the number of OTUs) (Figure 3). The Venn diagram shows that only 343 of the 3188 OTUs were shared among all three groups (Figure 4).

**Figure 5:**
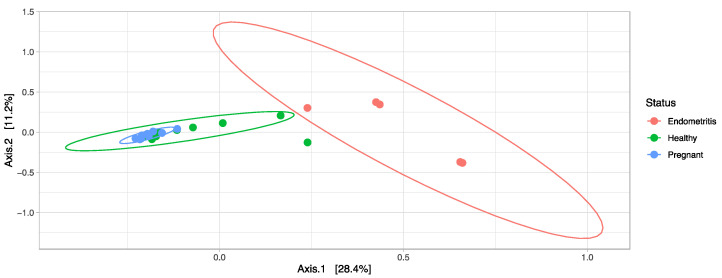
Principal component analysis (PCoA) based on Bray–Curtis dissimilarities of microbial community structure in uterine cytobrush samples taken ex vivo from 9 healthy cows, 10 pregnant cows and 5 cows diagnosed with endometritis.

As shown in the PCoA analysis, endometritic cows could be separated from healthy and pregnant cows (Figure 5). PERMANOVA analysis of the uterine samples confirmed significant differences in community composition between the endometritic animals and healthy (*p* = 0.003, R^2^ = 0.23) and pregnant animals (*p* = 0.003, R^2^ = 0.35), respectively. Three OTUs assigned to the genus *Escherichia*-*Shigella* (Enterobacteriaceae, Enterobacteriales; Gammaproteobacteria) were significantly more abundant in endometritic cows whereas one OTU assigned to the genus *Acetitomaculum* (Lachnospiraceae, Firmicutes) was significantly reduced (P_adj_ < 0.1). Between healthy and pregnant cows, 47 OTUs were found to be differentially abundant. Of these, only one OTU assigned to *Anaerobacillus* (Bacillaceae, Bacteroidetes) was significantly more abundant in pregnant cows (P_adj_ < 0.1) (Figure S1).

## 4. Discussion

In the present study, we investigated the bovine uterine microbiota following endometrial cytobrush sampling ex vivo in healthy dairy cattle, cattle with endometritis, and cattle in early stages of pregnancy. The animals were sampled at slaughter as to be able to collect the samples without animal welfare issues. Unlike other transcervical sampling approaches, we used samples directly taken from the uterus. This allowed us to take samples without the risk of vaginal or cervical contamination, as well as taking samples from pregnant animals. The latter would otherwise not be possible without abortion of the fetus. A similar study assessing the microbiome of the uterus of pregnant animals used the uteri post slaughter, though the sample taken was different. Unlike our study, they had to disinfect the outside of the uterus and as they took a full thickness uterine sample [19]. However, since in the pregnant uterus only low numbers of bacterial are present, it might have been better to disinfect the outside. Nevertheless, we took care of eventual contamination of the sample, using sterile material and not sampling at the incision place. Nevertheless, it should be taken into account that some of the OUTs detected might be contaminants. This study showed that the complexity and assemblage of the intrauterine bacterial community differs significantly between healthy cows and cows suffering from endometritis but seems comparable between healthy and pregnant cows. While the number of animals are rather low, especially the group of diseased animals, the data remain of interest as they add up to the gross data obtained on the uterine microbiome of cattle, especially those of the pregnant animals of which few data are available.

Microbial communities are typically more diverse in healthy endometria. In line with mounting evidence of commensal uterine colonization in the bovine reproductive tract, we also found that the uterus is not sterile even during pregnancy. However, few studies have been performed on the endometrial microbiome during pregnancy. Similar to those previous studies, the same—though with minor variations—microbial families are abundant in the bovine endometrium [11,31,32].

From the current results and also those found by other researchers, we have a more diverse microbiome in the healthy uterus, indicating that the diversity of species is necessary to have a healthy microbiome and maintain a healthy status. Disturbing factors, of which partus is one, may disturb this healthy microbiome and lead to the overgrowth of a pathogenic species. The ecological concept of species diversity in a healthy system has been demonstrated before [33]. More studies and data on the uterine microbiome and its evolution are necessary to demonstrate the relationship between lack of diversity and disease.

It was previously shown that different microbial populations are present in the non-pregnant endometrium compared to the embryo stage of pregnancy, though this was not found by all researchers and was only evident in the very early stages of pregnancy, close to the timepoint of insemination [10]. In our study, no differences between pregnant and non-pregnant healthy animals were observed. Both groups of animals clustered together in the PCoA analysis. It seems that there might not be a robust hormonal effect on the endometrial microbiome between these groups of animals as previously thought, though it should be taken into account that this study was only on animals in the early stages of pregnancy and thus we cannot exclude that a prolonged hormonal effect may have an effect. Moreover, the healthy and pregnant animals might have been more or less under the same progesterone effect as the pregnant animals were, only in an early stage of pregnancy. Our sample size was rather low and larger sample sizes and animals in different stages of reproduction can bring more clarity in this. The differences during early pregnancy might be a transient adaptation, or bacteria introduced with semen [30].

Differences between studies on healthy microbiomes thus show a large diversity; however, it has been shown in many different ecosystems that these differences in microbiomes are not related to the metabolic pathways they represent as a total. While the microbiomes may be different, the metabolic pathways may be the same [34]. This study adds up to our knowledge of the healthy microbiome of animals and will allow further studies to determine whether metabolic pathways are similar between the animals.

In postpartum animals, the microbiome shows an evolution starting with an alteration of the microbiome, then returning to normal after 5 weeks [14]. It is clear that microbial influx into the reproductive system of cows during calving is unavoidable and it has been shown that the vaginal flora shows similar changes [31,35]. However, some studies have failed to show an evolution in the postpartum microbiome unless there was subsequent endometritis, while other studies have shown a positive effect of the presence of certain bacterial species on fertility [32,36]. Some other studies failed to demonstrate the association of certain bacterial species with fertility [37]. Thus, it may be that there is a core endometrial microbiome present in both healthy and pregnant animals that maintains homeostasis and prevents disease. Further investigations of the role of hormones on the endometrial microbiome are warranted.

We found major differences between endometrial microbiomes of healthy and diseased cattle, even with a small sample. Both the alpha- and beta-diversity measures are very different. Presence of pathogenic bacteria becomes evident as some OTUs, which were identified to genus level, become dominant. There is also a major shift in the ‘normal’ microbiome. This is in line with earlier findings [32,38]. From our data, different bacteria are involved in disease processes, as it was possible to segregate the animals into three different ‘disease profiles’. However, it is crucial to sample endometrial microbiomes from a wide range of animals at different stages of uterine disease to have meaningful baseline data. Based on the most common bacteria isolated from endometritis in cattle [39], it is reasonable to assume that the probable infecting species could be *Trueperella pyogenes* (Actinobacteria), *Escherichia coli*/*Klebsiella pneumoniae* (Proteobacteria), and an infection with anaerobes which could be *Fusobacterium necrophorum* and *Bacteriodes* spp., as well as *Prevotella* species.

Using deep-level 16S rRNA amplicon-based gene sequencing several studies showed the involvement of different bacterial families and genera. The involvement of the *Peptostreptococcus* spp.; *Sneathia* spp.; *Prevotella* spp.; *Arcanobacterium* spp.; *Corynebacterium* spp.; Fusobacteriaceae, Mycoplasmataceae, and *Ureaplasma* spp.; and Bacteroidaceae, Porphyromonadaceae, and Pasteurellaceae families was shown [40,41,42], which is in part different from our study. We found mollicutes only in healthy cows (at low relative abundancy though) along with Pasteurellaceae, with the predominance of *Histophilus*, a typical genital-mucosa-dwelling bacterium which is a facultative pathogen in cattle. It is evident that cows, from different locations, with different types of endometritis need to be investigated to see patterns of infection. The uterine microbial signatures detected could ultimately lead to new diagnostic methods that allow more targeted treatments. However, given the sample size here, there is a need for more detailed studies comparing uterine microbial communities retrieved from endometritic animals, so potential microbial indicators for etiology, dysbiosis, and inflammation can be well defined and associated with a certain treatment and prevention program for PPUD.

In addition, metagenomic studies have been applied for studying the differences in uterine microbial composition between healthy and diseased animals. Here, the most prevalent species in diseased animals differed between the different studies and mostly members of the *Bacterioidetes* were found, while there was a negative association found for *Fusobacteria* and *Treuperella* [43]. Metagenomics also allows us to find associations with certain virulence genes, while health is associated with the presence of bacteriocins and antimicrobial peptides [43], also indicating the probiotics containing these products may be helpful.

We also detected a subclinical case of PPUD in this study for which the microbial alterations were already present. However, other studies, using different methodologies, could not find any association of subclinical metritis with certain pathogens [44,45]. Studying in detail many more (sub) clinical case and starting sampling from partus onwards to determine shifts in trends may predict which cows will develop PPUD. However, these studies will require larger cohorts of animals from different environments.

While there is an overrepresentation of pathogenic bacteria in diseased animals, there remains a part of the core genome that is important for recovery. Comparing this pathogen-associated ′residual core′ with the ′healthy core′ may lead to new insights in the role of the endometrial microbiome in preserving uterine health and lead to the development of probiotics and prebiotics that may fortify the core microbiome at partus and, as such, prevent the pathogen to get established.

It is thus important to know the details of the microbiome to be able to determine the health status of the animals, as well as to develop scientific based pre- and probiotics. Knowing the microbiome will help in the identification of animals at risk and will aid in the treatment. For the practitioner and farmer, it is also of interest to know that the uterus has its own microbiome and disturbances by any action may lead to metritis. Any manipulation (at oestrus synchronisation, or insemination or assisted calving or other) induce changes to this microbiome and may cause endometritis. The endometrial microbiome thus plays a very important role in uterine health by helping to control infection. We noticed reduced diversity with increased abundance of bacteria from genera that have been correlated with postpartum endometritis. It will also allow the farm veterinarian, farmer, or farm staff, to identify at risk animals and will allow use of pre- and/or probiotics, and therefore reduce use of antibiotic treatments.

## 5. Conclusions

Our data have shown that the uterine microbial communities are seemingly not influenced by pregnancy, though they form a separate group overlapping the healthy animals. Compared to the healthy microbiome, we noticed a reduced abundance and increase of certain bacterial genera, which is typical for postpartum endometritis microbiomes. These pattern-like changes may offer the possibility for developing new diagnostic tools that may allow a more targeted treatment of uterine disease. Further studies are necessary to confirm this. Comparison of microbial communities of the total genital tract, as well as analyzing in detail the microbial communities of cows with endometritis, may bring insights in the dynamics of disease. Through additional studies, also including culture-based methods, may offer opportunities to develop probiotics and/or prebiotics that may change these dynamics into a healthy direction.

## Data Availability

Sequencing data associated to this study are available in the Sequencing Read Archive (SRA) repository, BioProject ID: PRJNA942289, and accession numbers SAMN33683810-SAMN33683833.

## Supplementary Materials

The following supporting information can be downloaded at: https://www.mdpi.com/article/10.3390/vetsci10030215/s1, Figure S1: DESeq2 analysis with Wald test indicating the fold-change of bacterial genera in uterine bacterial communities of cows diagnosed with endometritis relative to healthy cows.
