# Supplementary material for: The Endometrial Microbiota—16S rRNA Gene Sequence Signatures in Healthy, Pregnant and Endometritis Dairy Cows
Source: Vet Sci. 2023 Mar 10;10(3):215. doi: 10.3390/vetsci10030215 (PMC10058826; doi:10.3390/vetsci10030215)
Supplement: Supplementary file 1 [file vetsci-10-00215-s001.zip › vetsci-2227839-supplementary.pdf]

A

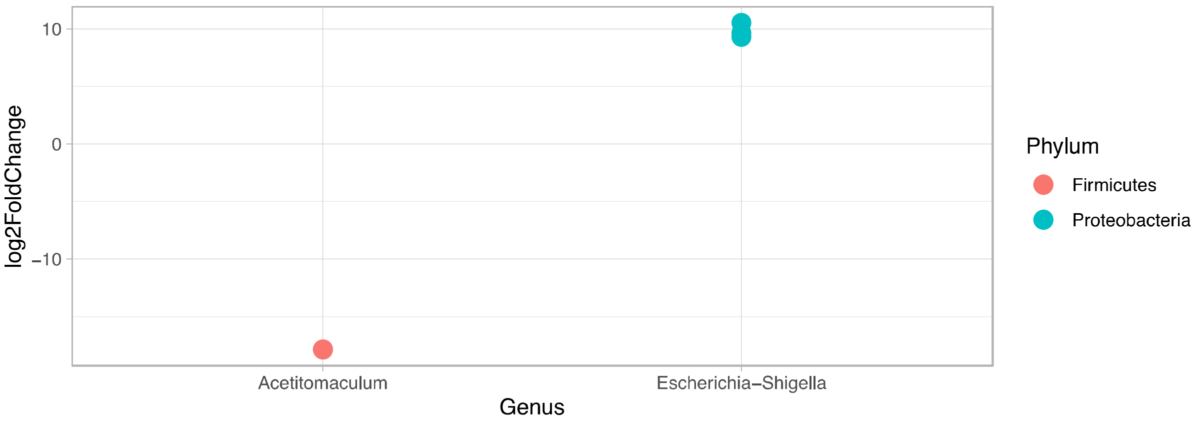

B

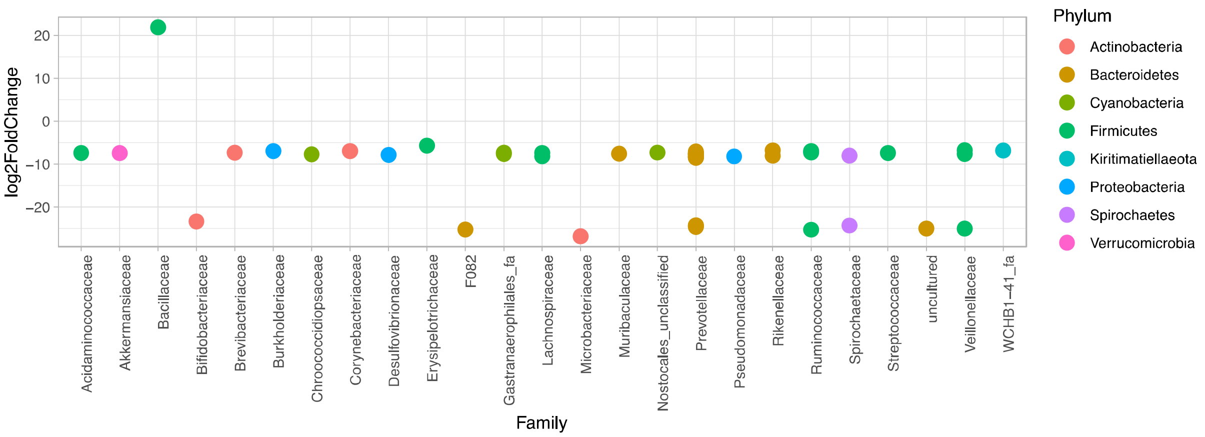

Figure S1. (A) DESeq2 analysis with Wald test indicating the fold-change of bacterial genera in uterine bacterial communities of cows diagnosed with endometritis relative to healthy cows ($P_{adj} < 0.1$). A positive fold change implies an increase of abundance in endometritic cows. **(B)** DESeq2 analysis results indicating the fold change of bacterial families in uterine bacterial communities of pregnant cows relative to healthy cows ($P_{adj} < 0.1$). A positive fold change implies an increase of abundance in pregnant cows.
